# The relationship between intellectual ability and auditory multitalker speech perception in neurodivergent individuals

**DOI:** 10.1371/journal.pone.0329581

**Published:** 2025-09-24

**Authors:** Bonnie K. Lau, Katherine Emmons, Ross K. Maddox, Annette Estes, Stephen R. Dager, Susan J. (Astley) Hemingway, Adrian K. C. Lee

**Affiliations:** 1 Virginia Merrill Bloedel Hearing Research Center, University of Washington, Seattle, Washington, United States of America; 2 Department of Department of Otolaryngology – Head and Neck Surgery, University of Washington, Seattle, Washington, United States of America; 3 UW Autism Center, University of Washington, Seattle, Washington, United States of America; 4 Department of Otolaryngology – Head and Neck Surgery, University of Michigan, Ann Arbor, Michigan, United States of America; 5 Department of Speech and Hearing Sciences, University of Washington, Seattle, Washington, United States of America; 6 Department of Radiology and Bioengineering, University of Washington, Seattle, Washington, United States of America; 7 Departments of Epidemiology and Pediatrics, University of Washington, Seattle, Washington, United States of America; 8 Institute for Learning and Brain Sciences, University of Washington, Seattle, Washington, United States of America; Father Muller Charitable Institutions, INDIA

## Abstract

The ability to selectively attend to one talker in the presence of competing talkers is crucial to communication in noisy, real-world environments. In this study, we investigated the relationship between intellectual ability and speech perception under multitalker conditions. Since neurodivergent individuals show a wide range of intellectual ability, from above average IQ to intellectual disability, intellectual ability may be an important individual characteristic that impacts multitalker speech perception, but this is not currently well understood. We tested individuals with autism, fetal alcohol spectrum disorder, and an age- and sex-matched comparison group, all with typical hearing. We found a strong positive correlation between IQ and multitalker speech perception thresholds. This demonstrates that deficits in intellectual ability, despite intact peripheral encoding of sound, are associated with difficulty listening under complex conditions for individuals with autism and fetal alcohol spectrum disorder. Future research is needed to investigate specific cognitive control mechanisms that contribute to difficulty listening under complex conditions. These findings suggest that audiological services to improve communication in real-world environments for neurodivergent individuals should be considered during clinical evaluations.

## Introduction

Multitalker speech perception, the ability to selectively attend to one talker in the presence of several competing talkers, is a crucial yet often underappreciated skill employed in everyday life. To purchase a drink in a coffee shop with loud background noise, one must selectively tune-in to the barista’s voice while filtering out the voices of other customers. To learn in a classroom, a student needs to ignore the voices of other students to focus on the teacher’s voice. In real-world settings involving multiple speakers, neurotypical listeners with typical hearing can effortlessly attend to the desired talker even when competing voices are significantly louder [1]. The ability to organize and extract meaning from competing sounds in these complex acoustic environments is often referred to as auditory scene analysis or the cocktail party problem [2].

Successful auditory scene analysis relies first on the accurate encoding of the incoming sound mixture by the ear and auditory nerve fibers, followed by the integration of sounds between the ears [see 3 for conceptual model]. These spectro-temporal patterns encoded by the auditory periphery are then segregated and grouped into separate auditory streams based on acoustic features, such as pitch or spatial cues, that indicate where the sound is coming from [4–7]. From there, more complex processing of the lexical, syntactic, and semantic information in the speech signal occurs [8,9]. Importantly, cognitive processes, including long and short-term memory, as well as attentional control, are thought to be required throughout both auditory grouping and complex processing stages [10]. The interplay between these cognitive and auditory perceptual processes are often modelled as complex functions, with involvement of both feedback and feedforward mechanisms [3]. Moreover, there is evidence that the selective enhancement of relevant speech is influenced by an attention-mediated feedback loop [3,11–14]. In the presence of multiple simultaneous talkers, listeners must also selectively direct their attention to the speaker of interest [6,15]. Many aspects of the relationship between cognitive and auditory processes, including how they function during multitalker speech perception, are not fully understood. However, attention and memory play a critical role in all stages of speech perception under complex conditions [3].

Auditory processing differences, including hyper- and hypo-sensitivity to sound, aversions to sound, difficulty listening in noisy environments, are commonly reported by individuals with autism and fetal alcohol spectrum disorder [FASD; 16–19]. Autism is a developmental condition characterized by difficulties with social communication and interactions, as well as restricted interests and repetitive behaviors, according to the Diagnostic and Statistical Manual of Mental Disorders, Fifth Edition [20]. Autism is estimated to affect 1 in a 100 people globally [21] and 1 in 36 children in the United States [22]. FASD describes a range of physical, functional, and neurological outcomes associated with prenatal alcohol exposure. It is estimated to affect 8 in 1000 children globally [23]. On the most severe end of the spectrum, Fetal Alcohol Syndrome (FAS) is a permanent birth defect characterized by significant central nervous system (CNS) abnormalities, growth deficiency, and a unique cluster of three minor facial anomalies—small eyes, thin upper lip, and a flat philtrum [24]. The majority of children will not receive a diagnosis of FAS [25], but a diagnosis of FASD, which can be characterized by physical differences such as facial dysmorphias as well as cognitive challenges, such as deficits in working memory, learning, communication, and attention [24,26].

Difficulty understanding speech under noisy conditions is commonly reported by autistic individuals [e.g., 27,28]. While there are no prior studies measuring speech perception abilities in individuals with FASD, the studies conducted in autistic individuals report variable findings of whether speech perception deficits are indeed observed and if so, under what conditions [29–32]. Variability in the results of these studies may result from heterogeneity in the participants themselves. Since individuals with autism and FASD show a wide range of intellectual ability, from above average IQ to intellectual disability, intellectual ability may be an important individual characteristic that impacts multitalker speech perception, but this is not currently well understood for neurodivergent individuals.

Given that cognitive processes are known to be active in speech perception [33–35] and the results of prior studies show a relationship between intellectual ability and speech perception under complex conditions [e.g., 36–39], the goal of this study is to investigate whether deficits in intellectual ability is related to speech perception difficulty for neurodivergent individuals. We tested neurodivergent individuals with varying intellectual quotient (IQ) and with clinically normal hearing thresholds who belonged to one of three diagnostic groups: autism, FASD, and an age- and sex-matched comparison group. We use the term neurodivergent to refer to individuals who process, learn, or behave in ways that differ, but are not necessarily inferior to neurotypical individuals. The rationale for testing these two groups is that individuals with autism and FASD have a wide range of IQ, including individual with intellectual disability, and they commonly report difficulty perceiving speech under noisy conditions.

## Methods

### Participants

Forty-nine subjects ($\begin{array}{c} n\;=12 \end{array}$ Autism; $\begin{array}{c} n=10 \end{array}$ FASD, $\begin{array}{c} n=27 \end{array}$ Comparison) participated in the study (see Table 1 for demographic information). Twelve adults diagnosed with autism spectrum disorder (ASD) were recruited from a larger longitudinal study conducted at the University of Washington [40]. The original cohort consisted of 72 children diagnosed with autism between the ages of 3 and 4 years. Diagnoses of ASD, according to criteria from the fourth edition of the Diagnostic and Statistical Manual of Mental Disorders [41] were made by a licensed clinical psychologist or supervised graduate student using: 1) the Autism Diagnostic Interview-Revised [42], 2) the Autism Diagnostic Observation Schedule [ADOS; 43], 3) medical and family history, 4) cognitive test scores, and 5) clinical observation and judgment [see 40 for further details]. These participants were tested again at ages 6, 9, and 13–15 years. For this current study, forty-six participants from the original cohort were re-contacted and invited to participate in this current study. The remaining 26 were not contacted because they were already recruited for another study. Of the 46 participants contacted, 4 had moved out of state, 2 were not interested in participating, 1 could not be scheduled, 25 did not respond to phone calls or emails, and 2 did not meet our eligibility criteria of being able to speak in 3-word phrases. Thus, twelve autistic participants from the original cohort were enrolled in the study and tested at age 21–23 years of age (see Table 1 for sample demographics). This response rate was consistent with our expectations as the participants in this study were first recruited between the ages of 3 and 4 years and many families have moved, changed contact information, or were no longer interested in participating in research.

**Table 1:** Sample demographics.

	ASD	FASD	Comparison
**n**	12 4 female	10 6 female	27 13 female
**Age** Range SD	21.69 20.9-23.3 0.62	20.37 13.7-47.1 9.86	23.08 13.2-45.4 7.03
**Ethnicity**			
Not Hispanic or Latino	10	8	26
Hispanic or Latino	1	2	0
Other/NA	1	0	1
**Race**			
White	8	4	21
Asian	0	0	3
Native Hawaiian or Other Pacific Islander	1	0	0
American Indian/Alaskan Native	0	4	0
Black or African American	0	2	0
More than one race	3	0	3

Ten participants with FASD were recruited from a university based FASD diagnostic clinic. All participants had a diagnosis of FASD as determined by the 4-Digit Diagnostic Code [24,26], which is an interdisciplinary approach to diagnosis guided by the magnitude of expression of the four features of FAS: growth deficiency, FAS facial phenotype, CNS structural/functional abnormalities, and prenatal alcohol exposure. Two participants had a diagnosis of fetal alcohol syndrome, two with a diagnosis of static encephalography/alcohol exposed, and six with a diagnosis of neurodevelopmental disorder/alcohol exposed (see Table 2 for speech perception thresholds and intellectual ability by FASD diagnoses). In terms of degree of dysfunction, “static encephalopathy, alcohol exposed (SE/AE)” can be used to indicate a significant CNS dysfunction in the context of prenatal alcohol exposure, and the term “neurodevelopmental disorder, alcohol exposed (ND/AE)” can be used to indicate a mild-to-moderate CNS dysfunction in the context of prenatal alcohol exposure [24]. Nine of ten FASD participants were between the ages of 13 and 22; however, one adult aged 47 was also included in the study. The initial recruitment goal was for 12 participants with FASD to match the Autism group sample size, however, we were only able to recruit 10 participants.

**Table 2:** Multitalker speech perception thresholds (TMR in dB) and WASI-II Full Scale IQ (FSIQ-4) by FASD diagnoses for participants in the FASD group [24,26].

	n	TMR	WASI-II FSIQ-4
Fetal Alcohol Syndrome	2	4.5(0.34)	70.00(10.00)
Static Encephalography/Alcohol Exposed	2	3.53(2.75)	97.00(10.00)
Neurodevelopmental Disorder/Alcohol Exposed	6	−0.16(1.08)	106.00(6.37)

*Note:* Mean ± SE shown in parenthesis.

The Comparison group consisted of 27 participants. For each participant in the Autism and FASD groups, one age- and biological sex-matched participant was recruited as a pair-matched comparison subject. Age matching in comparison participants was ± 1 year except the 47-year-old participant in the FASD group was matched to a 45-year-old comparison subject. A one-way analysis of variance confirmed that there was no significant difference in age between groups (F(2,46) = 0.611, *p* = 0.547). Participants were also matched on biological sex due to the higher incidence of males with a diagnosis of autism. Besides the pair-matched comparison subjects, there were also an additional five Comparison group participants tested. These five additional participants were recruited for the magnetoencephalography component of the larger study. Comparison group participants all reported no history of cognitive, developmental, or other health concerns except attention-deficit/hyperactivity disorder (ADHD). As there is a high incidence of ADHD in individuals with FASD and autism, ADHD was not included in the exclusion criteria for any group. In our participant sample, 25% had parent-reported ADHD (Autism = 17%, Comparison = 8%, FASD = 80%). We found no evidence for a relationship between report of ADHD and performance on our multitalker speech perception task (further details in Discussion and Supporting Information). Additional exclusion criteria for all three participant groups included: a history of sensory or motor impairment such as hearing loss, traumatic brain injury, major physical anomalies, genetic disorders associated with ASD such as Fragile X, or other neurological impairments such as seizures.

### Stimuli

The multitalker listening environment was constructed from three sentences spoken simultaneously. The sentence stimuli were from the Coordinate Response Measure (CRM) corpus [44], which consists of 256 sentences of the form “Ready (CALLSIGN) go to (COLOR) (NUMBER) now.” There are eight possible call signs (Arrow, Baron, Charlie, Eagle, Hopper, Laker, Ringo, Tiger), four colors (red, green, white, blue) and the numbers 1–8. Two male and two female speakers speaking all combinations of the call signs, colors, and numbers were included in the stimulus set. Using custom Python software, head-related transfer functions (HRTFs) from the CIPIC HRTF database [45], were resampled to 24,414 Hz to match the stimulus sampling rate then convolved with the target and masker sentences to simulate the source locations tested in this experiment: the target stream to be attended at 0º azimuth and the two spatially separated masking speech streams at ± 45º azimuth. The target talker was always male, while the two maskers were either male/male or female/female. Sentences were randomly selected on each presentation.

### Procedures

The following measures were obtained over the course of several visits and as part of a larger study that included additional neurophysiological and behavioral measures. The number of visits for the larger study ranged from two to five, based on the preference of participants. In the present study, the Autism Diagnostic Observation Scale, Second Edition [ADOS-2; 46], and The Weschler Abbreviated Scale of Intelligence – Second Edition [WASI-II; 47] was administered on the same visit. For most participants, the audiological screening and the multitalker adaptive speech perception threshold task were obtained on a separate visit, but within the span of 4 weeks. Written informed consent was obtained from all participants or their Legally Authorized Representative for participants under 16 years of age. All methods were performed in accordance with the protocols approved by the Institutional Review Board at the University of Washington where the research was conducted. Participants were provided with monetary compensation for their time. An autistic community member was consulted during the preparation of this manuscript.

### Autism symptoms and categorization

The ADOS-2 [43], a measure of autism symptom severity, was administered to all participants at the time of testing to confirm group inclusion. All participants in the Autism group received a classification of autism or autism spectrum based on their performance on the ADOS-2 while all participants in the FASD or Comparison received a classification of non-spectrum.

### Intellectual ability

The Weschler Abbreviated Scale of Intelligence – Second Edition [47] was used as a measure of intellectual ability. The Block Design, Vocabulary, Matrix Reasoning, and Similarities subtests were administered to each participant to estimate their Full Scale IQ (FSIQ-4) or general intellectual ability. The Block Design subtest measures the ability to analyze and synthesize abstract visual stimuli. The Vocabulary subtest is designed to measure word knowledge and verbal concept formation. On the Matrix Reasoning subtest, the participants view an incomplete matrix or series and selects a response option that completes the matrix or series. This subtest measures fluid intelligence, broad visual intelligence, classification, spatial ability, knowledge of part-whole relationships, simultaneous processing and perceptual organization. Finally, the Similarities subtest measures verbal concept formation and reasoning. As a measure of verbal IQ, the Verbal Composite Index (VCI) score was computed from the Vocabulary and Similarities subtests and as a measure of nonverbal IQ, the Perceptual Reasoning Index (PRI) scores were computed from the Block Design and Matrix Reasoning subtests.

### Audiological screening

Typical hearing was an inclusion criterion to participate in this study. To ensure clinically normal hearing thresholds, all participants were required to pass an audiometric screen (≤20 dB hearing level at octave frequencies between 500 and 8000 Hz) as well as a distortion product otoacoustic emission (DPOAE) screen. For the DPOAE screening, DPOAE growth functions were estimated at the distortion frequency of 2f1-f2. The frequency of the f2 primary tone was 4 kHz and the frequency and level of the f1 tone were varied according to formula in Johnson et al. (2006) [48]. One participant with ASD was excluded from this study because they failed the audiometric screening.

### Multitalker adaptive threshold task

**Fig 1:**
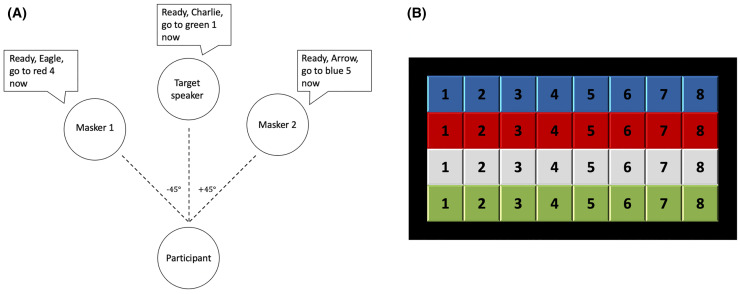
Schematic diagram of trial configuration and response panel. **A)** Participants heard three simultaneous sentences, with the target talker in front at 0° azimuth presented with two spatially separated simultaneous competing talkers, “maskers”, on each side at ±45° azimuth. **B)** Participants were asked to identify via mouse click the color and number spoken by the target talker on a computer screen.

To assess multitalker speech perception, participants listened to three simultaneous sentences and were asked to identify the color and number spoken by the target talker, who was always situated in front. The two competing talkers, who we refer to as “maskers,” were presented off to the side (Fig 1). Participants only heard the sentence stimuli (auditory only) and the spatial location of the talkers were simulated using head-related transfer functions. At the start of the task, the target talker is louder than the competing maskers which are presented at a much lower sound level. To increase task difficulty, we increased the sound level of the competing maskers adaptively to obtain a speech perception threshold for each participant.

These experimental procedures closely follow those outlined in Gallun et al. (2013) [49], except that stimuli were presented via insert ear tips in both ears as opposed to in sound field. Participants listened to three simultaneous sentences with the target stream to be attended was always positioned in front at 0º azimuth and two spatially separated masking speech streams at ± 45º azimuth. The goal was to attend to one of the three CRM sentences, identified by the callsign “Charlie,” and correctly identify the color and number spoken by the target talker. Speech perception thresholds, in terms of target-to-masker ratios (TMRs), were estimated using a one-up-one-down procedure, to estimate 50% correct [50]; the target level was fixed at 40 dB SPL and the masker levels were adaptively varied. The starting level for the masker sentences was 40 dB and they were changed in level by 5 dB for the first three reversals and then 1 dB for six additional reversals. Thresholds were estimated to be the geometric mean of the last six reversals. TMR is expressed as the level difference (dB) between the target and the two competing talkers, where a positive TMR indicated that the target had to be louder than the competing talkers, and a negative TMR indicated that the target talker would be quieter than the competing talkers. Thus, TMR scores in the positive range reflect poor speech perception performance, meaning that the listener needed the target talker to be louder than the maskers to accurately perceive the target talker’s speech. Each participant completed five adaptive tracks; the worst run (i.e., highest threshold value) was dropped and the best four runs were averaged.

To ensure audibility of the target sentence and to demonstrate the ability to perform the task, participants had to obtain 8 out of 10 trials correct on a training run where the target sentences were presented in quiet before testing began. If participants were not able to obtain 80% correct in quiet after a maximum of three attempts, they were excluded from the study; no participants were excluded for failing training. Feedback was provided for correct and incorrect trials only during the training run(s).

All auditory stimuli were presented via Etymotic ER-2 insert earphones in a sound-attenuated booth. Participants initiated each trial and indicated the color and number keywords associated with the target callsign “Charlie” by clicking a virtual button on a grid of color and number targets. All participants completed the five runs in a single session of about 30 minutes.

### Statistical analyses

To test the main hypothesis that deficits in intellectual ability are associated with poor speech perception thresholds, a simple linear regression was used to investigate whether WASI-II FSIQ-4 scores was significantly correlated with TMR. Three further analyses were conducted to assess the stability of this relationship. A simple linear regression was used to investigate whether intellectual ability was significantly correlated with speech perception thresholds within each group. Next, to investigate the relationship between verbal and non-verbal abilities, paired sample t-tests were first used to test for differences in average VCI and PRI scores within each diagnostic group. A simple linear regression was then used to investigate the relationship between verbal (VCI) or non-verbal ability (PCI) and speech perception. Finally, a simple linear regression was used to investigate the relationship between speech perception and performance on each individual WASI-II subtest. All tests were evaluated against at two-tailed *p* < 0.05 level of significance. Pair matching was conducted during the recruitment of the Comparison group participants to achieve a balance in age and sex between the study groups. This pair matching was not maintained in the statistical analyses because preliminary analyses conducted using pair matched linear regression with Autism and FASD groups and their respective Comparison groups revealed no effect of age; thus, all 27 Comparison participants were combined into one group. The total sample size of 49 was sufficient to detect the relationship between speech perception and intellectual ability using a one-sided test with a significance level of 0.025 to test whether the observed slope of −0.68 is greater than zero with a power (1-β) of > 0.99.

## Results

### Multitalker speech perception as a function of IQ

**Fig 2:**
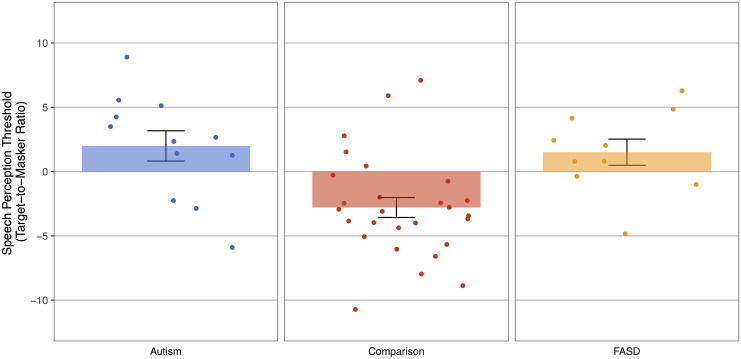
Speech perception thresholds (TMR at 50%) as a function of group. The Autism group is in blue (left), the Comparison group is in red (middle), and the FASD group is in orange (right).

**Fig 3:**
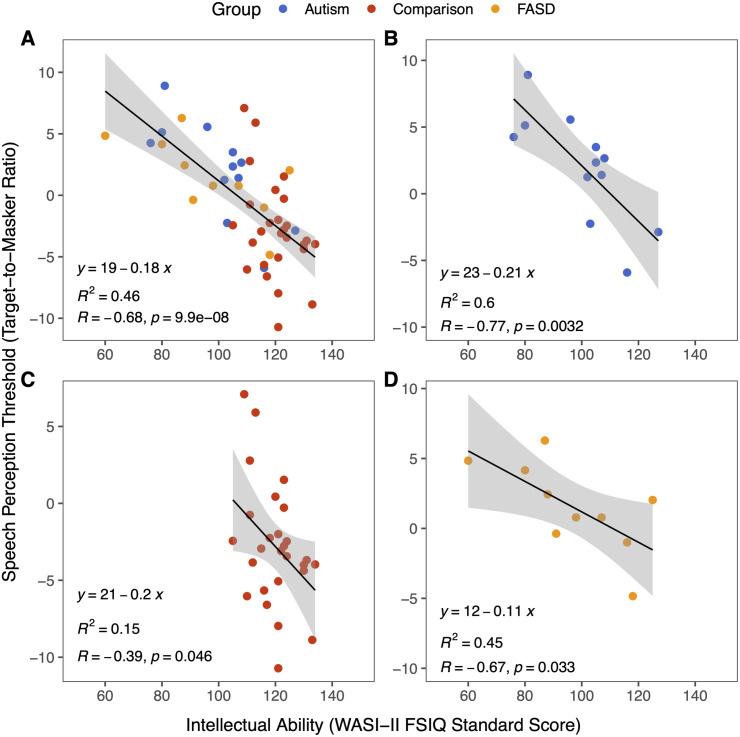
Speech perception performance improves with higher intellectual ability. **A)** Individual speech perception thresholds (TMR at 50% correct) as a function of intellectual ability (WASI-II FSIQ-4), with Autism group in blue, Comparison group in red, and FASD group in orange. Negative TMRs reflect better speech perception performance. Intellectual ability was correlated with TMR in the overall sample. **B-D)** Speech perception thresholds as a function of intellectual ability for the Autism **(B)**, Comparison **(C)**, and FASD **(D)** groups. Individual data points shown with solid circles. FSIQ-4 was correlated with TMR in Autism, Comparison, and FASD groups.

Multitalker speech perception thresholds across groups are shown in in Fig 2 and speech perception thresholds as a function of intellectual ability are shown in Fig 3. Lower WASI-II FSIQ-4 scores were related to higher speech perception thresholds in the overall sample (Fig 3A; linear regression, $\begin{array}{c} \beta =-0.68,t=-6.29,p<0.0001,R^{\text{2}}=0.46,\eta^{\text{2}}=0.46); \end{array}$ As intellectual ability decreased, speech perception performance (the ability of the listener to selectively attend to one talker in the presence of maskers) also decreased. We then investigated whether the relationship between intellectual ability and speech perception thresholds held within each diagnostic group. Indeed, intellectual ability (FSIQ-4 score) was significantly correlated with speech perception thresholds in all three groups (Fig 3B-D; linear regression, Autism, n = 12: $\begin{array}{c} \beta =-0.77,t=-3.85,p=0.003,R^{\text{2}}=0.60,\eta^{\text{2}}=0.60; \end{array}$ Comparison, n = 27: $\begin{array}{c} \beta =-0.39,t=-2.10,p=0.046,R^{\text{2}}=0.15,\eta^{\text{2}}=0.15 \end{array}$; FASD, n = 10: $\begin{array}{c} \beta =-0.67,t=-2.57,p=0.033,R^{\text{2}}=0.45,\eta^{\text{2}}=0.45 \end{array}$). However, the strength of the correlation is lower for the Comparison group than for the Autism and FASD groups.

During secondary analyses, the presence of influential outliers was assessed using Cook’s distance. Three influential outliers were identified and thus, a clean model with the outliers removed was conducted. Comparing the coefficients between the original versus clean model revealed that removing influential observations did not meaningfully alter the model. Both the slope (−0.182 to −0.181) and intercept (19.41 to 19.39) changed only slightly, suggesting that the findings of the original model are robust. An additional multivariate linear regression model was also conducted to control for biological sex, age, and participant group. While FSIQ-4 score remained a significant predictor of speech perception thresholds (multiple linear regression, $\begin{array}{c} \beta =-0.17,t=-4.41,p<0.0001,R^{\text{2}}=0.49,\eta^{\text{2}}=0.47) \end{array}$, age, sex, and group were not statistically significant (*p* < 0.05 for all). These results suggest that in our study sample, age, sex, and participant group did not have a significant effect on the relationship between IQ and multitalker speech perception.

### Verbal and non-verbal abilities

**Fig 4:**
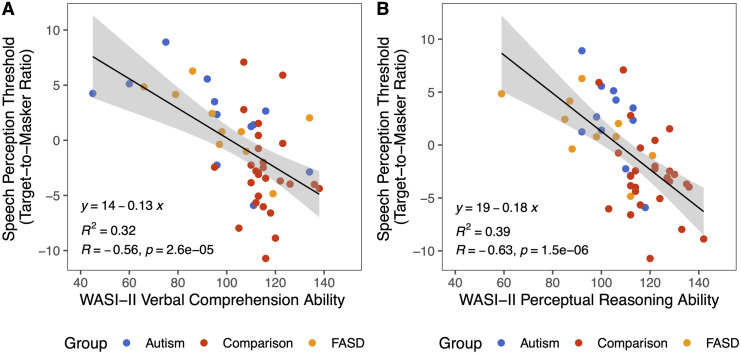
Speech perception performance correlated with both verbal and nonverbal abilities. Individual data points shown with solid circles. **A)** Verbal comprehension as a function of multitalker speech perception performance. WASI-II VCI was significantly correlated with TMR. **B)** Perceptual reasoning ability as a function of multitalker speech perception performance. WASI-II PRI was significantly correlated with TMR.

One possibility for these findings is that verbal ability is driving the relationship between the WASI-II FSIQ-4 scores and speech perception thresholds. To further investigate, we tested the relationship between verbal and non-verbal abilities with the Verbal Comprehension Index score (VCI) and the Perceptual Reasoning Index score (PRI) of the WASI-II. We conducted paired-samples t-tests to compare average VCI and PRI scores within each diagnostic group. We did not observe a difference between the two scores in any of the three groups (Autism paired $\begin{array}{c} t(11)=-1.35,p=0.21;\text{Comparisonpaired}t(26)=-1.67,p=0.11;\text{FASD paired}t(9)=0.89,p=0.4). \end{array}$ Furthermore, both WASI-II VCI (Fig 4A, linear regression, $\begin{array}{c} \beta =-0.56,t=-4.66,p<.0001,R^{\text{2}}=0.32,\eta^{\text{2}}=0.32 \end{array}$) and WASI-II PRI were correlated with speech perception performance in the overall sample (Fig 4B, linear regression, $\begin{array}{c} \beta =-0.63,t=-5.51,p<0.0001,R^{\text{2}}=0.39,\eta^{\text{2}}=0.39) \end{array}$, with comparable strength and effect size. The higher the VCI and PRI scores, the better the speech perception performance, showing that the relationship holds for both verbal and nonverbal abilities; thus, ruling out the possibility that verbal ability alone drove the relationship.

### Individual subtests

**Fig 5:**
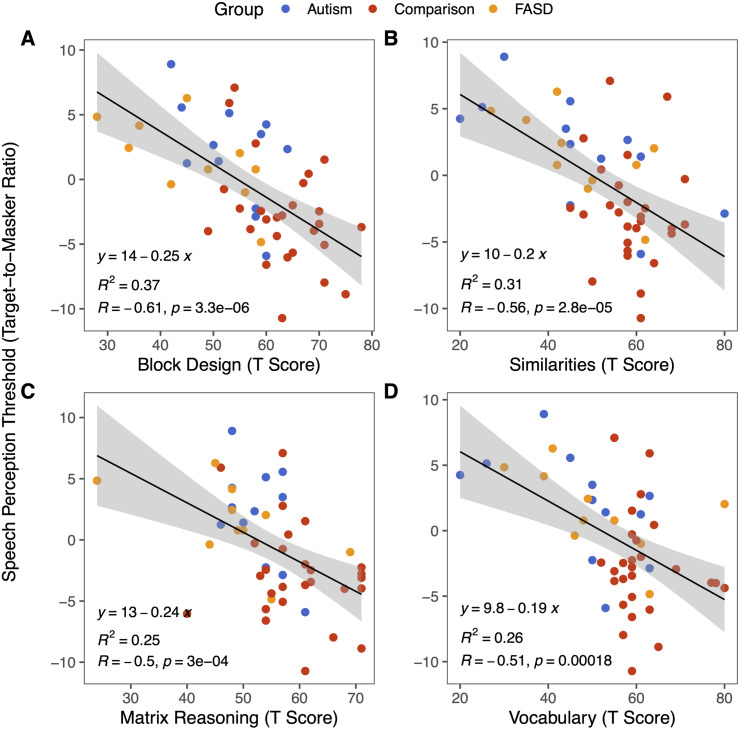
Speech perception performance correlated with performance on all subtests of the WASI-II. Individual data points shown with solid circles. **A)** Block Design subtest T Score as a function of TMR. **B)** Similarities subtest T Score as a function of TMR. **C)** Matrix reasoning subtest T Score as a function of TMR. **D)** Vocabulary subtest T Score as a function of TMR.

Each WASI-II subtest measures different intellectual abilities, so it also remains possible that multitalker speech perception is related to the specific intellectual abilities measured in some subtests but not others. To further investigate, we tested the relationship between speech perception thresholds and performance on each subtest of the WASI-II for the overall sample. Mean WASI-II subtest T scores for each participant group are shown in Table 3. On average, both Autism and FASD groups had lower T scores than the Comparison group for all four subtests. However, the FASD group showed more variability in performance in all four subtests as well, while the Autism group showed more variability than the Comparison group on the Similarities and Vocabulary subtests. We found a significant correlation between the speech perception thresholds and each of the four subtests: Block Design (Fig 5A, linear regression, $\begin{array}{c} \beta =-0.61,t=-5.27,p<.001,R^{\text{2}}=0.36,\eta^{\text{2}}=0.37 \end{array}$), Similarities (Fig 5B, linear regression, $\begin{array}{c} \beta =-0.56,t=-4.65,p<.001,R^{\text{2}}=0.30,\eta^{\text{2}}=0.31 \end{array}$), Matrix Design (Fig 5C, linear regression, $\begin{array}{c} \beta =-0.49,t=-3.91,p<.001,R^{\text{2}}=0.23,\eta^{\text{2}}=0.25 \end{array}$), and Vocabulary (Fig 5D, linear regression, $\begin{array}{c} \beta =-0.51,t=-4.07,p<.001,R^{\text{2}}=0.25,\eta^{\text{2}}=0.26 \end{array}$), with comparable strength and effect size for each subtest. In all cases, the higher the scores, the better the speech perception performance, showing that the relationship holds also for each individual subtest.

**Table 3:** Mean WASI-II subtest T scores by group with standard deviation shown in parenthesis.

	Block Design	Vocabulary	Similarities	Matrix Design
Autism	53.67(7.25)	47.75(13.61)	47.17(16.92)	52.67(4.73)
Comparison	63.37(7.33)	61.40(7.03)	58.74(6.81)	59.56(7.85)
FASD	46.2(10.99)	51.20(14.24)	47.40(12.04)	48.60(11.20)

## Discussion

We observed an association between intellectual ability and multitalker speech perception thresholds; as intellectual ability increased, multitalker speech perception thresholds improved. This relationship was not driven by verbal abilities alone as the correlation between speech perception performance held for both the VCI and the PRI. Moreover, the correlation between multitalker speech perception thresholds was also observed for each individual WASI-II subtest, suggesting that performance on the speech perception task is indeed related to general intelligence. Although we did not have a sufficient sample size to statistically test group differences on average, the Autism and FASD groups required the target speaker to be louder than the two competing talkers, as reflected by positive target-to-masker ratios. In contrast, on average, individuals in the Comparison group were able to selectively attend to the target talker even when competing voices were louder, although some individuals did have positive target-to-masker ratios. These results are consistent with prior studies showing worse speech perception in autistic adults and children [29,30,32]. They are also consistent with self/caregiver reports of auditory processing differences including difficulty listening to speech under noisy conditions in individuals with FASD and Autism [18,51–55]. To our knowledge, this is the first study to obtain multitalker speech perception thresholds in individuals with FASD.

A strength of this study is that we considered several characteristics of individuals with Autism and FASD in designing our study and analyses. First, we conducted an audiometric screen at octave frequencies between 250–8000 Hz (≤20 dB Hearing Level), as well as an otoacoustic emissions screen, to ensure clinically normal hearing thresholds in all participants, as there is an increased incidence of hearing loss in both populations [18,56–58]. Second, we chose a receptive speech perception task in which participants did not require verbal responses, as our intent was to assess speech perception rather than speech production. Third, we chose an adaptive threshold task, so that task difficulty would be varied systematically based on individual performance. Importantly, an adaptive threshold task design ensured that participants were able to perform the speech perception task when conditions were favorable (i.e., target talker is louder than the distractors).

The relationship between intellectual abilities and multitalker speech perception was observed in all participant groups, including the Comparison group. Listening in noisy environments is known to be difficult for individuals who are hard of hearing, with hearing assistive technology and auditory rehabilitation therapy often employed to address this challenge. However, this study suggests that individuals with typical hearing but lower intellectual ability may also have trouble listening under complex acoustic conditions. This suggests that those children who are intellectually challenged and the furthest behind their peers will have the most need to learn in noisy classrooms but, may also have the most trouble extracting information in complex listening environments. Consequently, future studies are needed to develop targeted assessment and intervention strategies for speech perception difficulties among neurodivergent individuals living in noisy real-world conditions.

The WASI-II is the gold-standard measure of general intellectual ability; however, it does not provide information on the specific cognitive mechanisms involved in multitalker speech perception. We ruled out the possibility that verbal abilities are driving the relationship and also showed that speech perception is related to each of the individual subtests, suggesting that it is in fact general intellectual ability that is related to the multitalker speech perception task employed in this study. While we did see the relationship to each individual subtest, future research is needed to determine why. For example, how are the abilities measured by matrix reasoning related to complex speech perception? Future research also needs to employ distinct cognitive tasks to study the mechanisms that play a role in complex listening as this current study was not designed to identify specific cognitive mechanisms.

In this study, we demonstrate that general intellectual ability is related to multitalker speech perception. However, we previously reported no effect of intellectual ability on stream segregation and selective attention in autism or a comparison group [59]. One possible explanation for these divergent findings is the difference in cognitive load between the tasks [60]. In Emmons et al. we presented a dual stream selective attention task with two talkers who each spoke two words. In this current paradigm, there are three simultaneous talkers speaking full sentences, that requires greater listening effort. For individuals with higher intellectual ability, this increased cognitive load may not have a discernable effect on complex listening. For individuals with lower ability, however, the effort associated with segregating and selectively listening in a multitalker situation may be sufficient to reduce their ability to remember a color and a number that was spoken by the target speaker. This may be especially true for individuals with FASD, who often experience deficits in working memory. Moreover, this conjecture is consistent with the fact that all participants in this study, regardless of ability, were able to perform the multitalker speech perception task with positive TMRs when the target talker was louder than the competing talkers.

There are several limitations to consider when interpretating findings from the present study. First, FASD and ASD are both heterogenous populations and the sample size in this study is small [61]. Future studies with larger cohorts will be required to further investigate multitalker speech perception differences between FASD, Autism, and Comparison groups, while controlling for IQ and to characterize individual variability. Second, the primary goal of this study was to investigate whether difficulty with multitalker speech perception is observed with deficits in intellectual ability, in the absence of hearing loss. The challenge with this study design was to determine participant groups that did not have hearing loss but did have variability in intellectual ability. Thus, we recruited from two groups that met these criteria: autism and FASD, along with a matched comparison group. While we had strict exclusion criteria to rule out most co-morbid health and neurological concerns, with the high incidence of attention deficit in FASD and autism, it was not possible for ADHD to be an exclusion criterion in this participant sample. Thus, we obtained a medical history from each participant or their parent/guardian prior to participation and conducted an additional analysis on whether ADHD status affected their performance on our multitalker speech perception task. We found no evidence for a relationship between report of ADHD and performance on our task (S1 Fig; see Supporting Information for details of the analysis). Nonetheless, an important limitation to note in this study is that for the participants that reported ADHD, we cannot determine the contributions of attention deficit to task performance, although at group level, no statistically significant difference was observed. Moreover, while an ADHD diagnosis in combination with autism or FASD did not affect performance on this particular speech perception task, it is unlikely the case for all complex speech perception scenarios. In fact, whether ADHD impairs complex speech perception is still in debate, with conflicting findings from different studies, with some reporting worse speech perception in individuals with ADHD but not others [62–64]. Future studies designed to assess both attention and complex speech perception are required to evaluate how attention deficits in addition to autism or FASD could impact multitalker speech perception. The critical point to note is that regardless of ADHD status, all participants were screened for clinically normal-hearing thresholds, and thus, our results demonstrate that the multitalker speech perception difficulty observed was not caused by peripheral deficits in sound encoding and had a strong association with intellectual ability. These results provide novel evidence that listening in complex situations may be impaired in individuals with lower IQ, with or without ADHD.

This study attempted to create a real-world listening scenario using full sentence stimuli and multiple speakers talking at the same time. While we simulated a realistic multitalker situation in the laboratory, there are many possible differences that may arise in situ, in the face of a multisensory environment. Future studies should consider how neurodiverse individuals listen in the context of non-human sounds alongside multiple talkers, as well as a range of multisensory input such as smells, lighting, and a visually crowded environment. Another aspect of multitalker speech perception is the communication demands on the listener. For example, a student in a lecture may be focused on listening; however, in a seminar, they would not only be expected to listen but also to participate by responding. Speech perception in situ might also be affected by both speaker behavior and expectations, guided by eye contact, nodding, and even verbal manipulation of attention. Besides these intricate dynamics of social interaction, speaker interest and motivation in the conversation topic as well as the mode of communication also contribute to speech perception in the real world.

In conclusion, in this investigation of multitalker speech perception in neurodivergent individuals with typical hearing, we found a highly significant relationship between directly assessed intellectual ability and multitalker speech perception. This relationship was observed within each diagnostic group separately, suggesting this effect may cross diagnostic boundaries. This is one of the first studies to investigate multitalker speech perception in individuals with FASD. From a clinical perspective, these findings suggest that assessment of complex listening ability and support for listening under complex, real-world environments may improve outcomes for neurodivergent individuals with lower cognitive ability. Finally, the findings raise the question of whether there could be shared etiology between multitalker speech perception and intellectual ability, genetic or neurologic, versus a causal relationship between the two.

## Supporting information

S1 Fig Multitalker speech perception thresholds as a function of ADHD status. (TIF)
