# Supplementary figures and images for: The relationship between intellectual ability and auditory multitalker speech perception in neurodivergent individuals
Source: PLoS One. 2025 Sep 24;20(9):e0329581. doi: 10.1371/journal.pone.0329581 (PMC12459794; doi:10.1371/journal.pone.0329581)

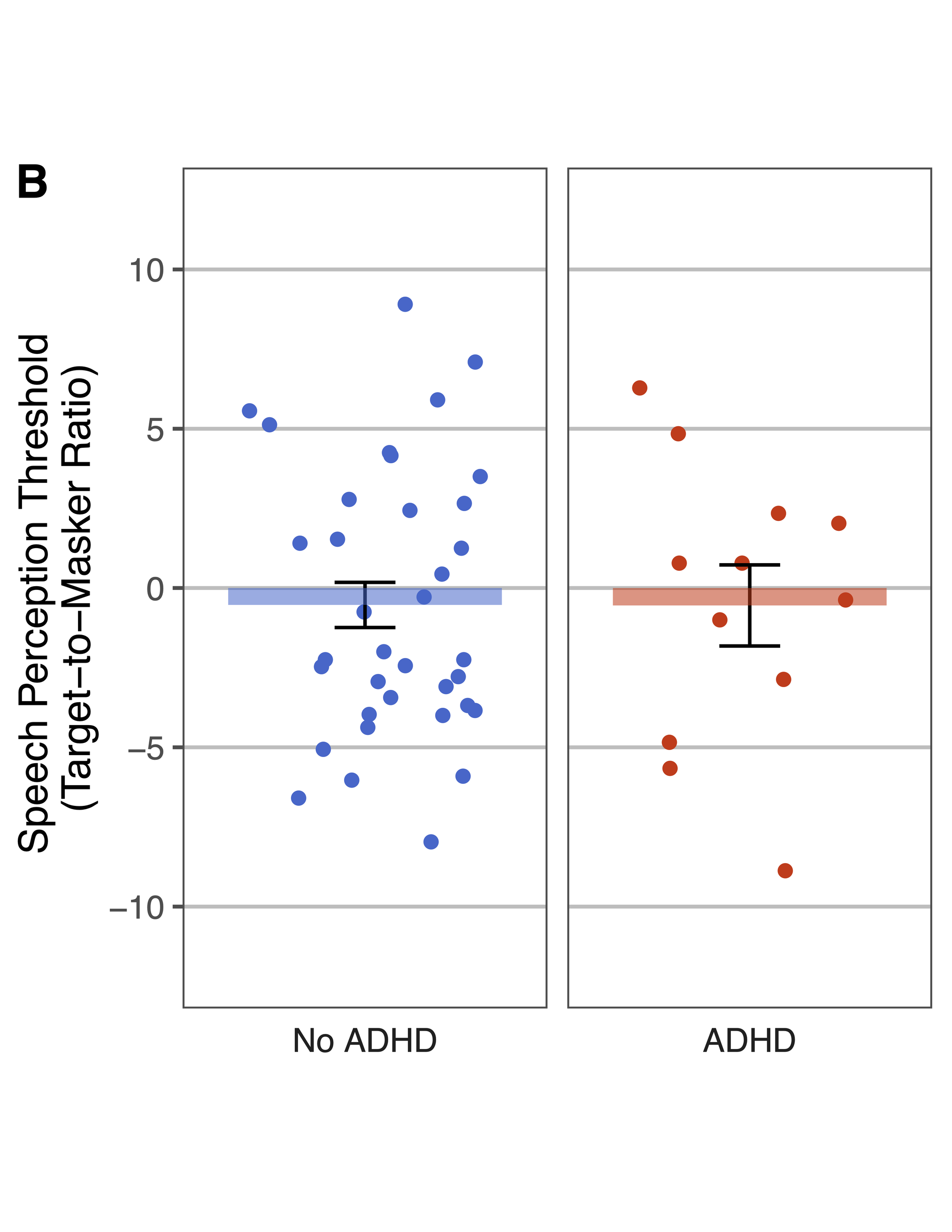

Supplement: S1 Fig — (TIF) [file pone.0329581.s001.tif]
